# Targeting PI3Kα with Small Molecules: Progress and Challenges in Tumor Treatment

**DOI:** 10.3390/cimb48100968

**Published:** 2026-09-22

**Authors:** Wenqing Jia

**Affiliations:** College of Chemistry and Chemical Engineering, Qilu Normal University, Jinan 250200, China; wqj0312@qlnu.edu.cn

**Keywords:** PI3Kα, PI3Kα inhibitor, tumor, clinical trial, drug development

## Abstract

Aberrant activation of PI3Kα tightly correlates with tumorigenesis and advancement. PI3Kα inhibitors can bring significant clinical gains to patients with *PIK3CA*-mutant breast cancer and other solid tumor patients by inhibiting tumor cell growth and regulating the immune microenvironment. Alpelisib, a selective PI3Kα inhibitor, has a more favorable benefit-to-risk ratio compared with earlier-generation pan-PI3K inhibitors, with more manageable risks of severe adverse events, including pneumonitis. CYH33 induces immune activation and synergizes with a FASN inhibitor to further promote antitumor immunity. In addition, inavolisib, which features a mutant-selective degradation mechanism, has already been approved for clinical use, while compounds with distinct mechanisms, such as the wild-type PI3Kα-sparing allosteric inhibitor STX-478, have demonstrated favorable preclinical results and are currently under phase I/II clinical research for *PIK3CA*-mutated advanced solid tumors. Apart from the above therapies, emerging drugs with differentiated mechanisms have shown great potential for clinical application in the years ahead. BEBT-908 not only inhibits the growth of tumor cells but also activates host anti-tumor immunity to strengthen immune checkpoint treatment. Additionally, ZM-PI05 represents a PROTAC agent that degrades the PI3Kα protein. Despite the evident clinical advantages of PI3Kα inhibitors, toxicities such as hyperglycemia, rash, and gastrointestinal reactions limit their popularization. Current research mainly focuses on designing selective PI3Kα inhibitors with improved safety and establishing optimal medication regimens. This paper discusses the biological roles of PI3Kα in tumors, summarizes clinical trials, common side effects, and binding characteristics of PI3Kα inhibitors, and proposes guidance for their further development and utilization.

## 1. Introduction

Phosphatidylinositol 3-kinase α (PI3Kα) is a crucial intracellular lipid kinase belonging to the class I phosphatidylinositol 3-kinase family, which functionally exists as a heterodimer in mammalian cells, consisting of the *PIK3CA*-encoded catalytic p110α subunit and the regulatory p85α subunit. As a key intracellular kinase that targets phosphatidylinositol substrates, PI3Kα exerts its core enzymatic activity through catalytic phosphorylation reactions. Under physiological conditions, activated PI3Kα specifically phosphorylates phosphatidylinositol 4,5-bisphosphate (PIP_2_) on the cell membrane to generate phosphatidylinositol 3,4,5-trisphosphate (PIP_3_), an essential lipid secondary messenger in cells [1,2]. The accumulated PIP_3_ further recruits and activates a variety of downstream signaling proteins, predominantly triggering the phosphorylation and activation of the protein kinase B (Akt)/mammalian target of rapamycin (mTOR) signaling cascade [3]. This process modulates multiple fundamental cellular biological processes, including cell proliferation, apoptotic inhibition, cell cycle progression, energy metabolism, and cell differentiation [3]. The precise regulation of the PI3Kα-mediated signaling axis is indispensable for maintaining normal cellular physiological homeostasis and tissue function.

Accumulating preclinical and clinical evidence has demonstrated that aberrant activation and dysregulated expression of PI3Kα are closely correlated with the initiation, malignant progression, metastasis, and drug resistance of multiple solid tumors, as well as the occurrence and deterioration of chronic metabolic diseases. Accordingly, PI3Kα has become a critical target for mechanistic research and clinical targeted therapy of cancers and metabolic disorders [3,4]. Pathologically, the persistent abnormal activation of PI3Kα is predominantly driven by somatic gain-of-function mutations in the *PIK3CA* gene. These hotspot mutations are mainly located in the helical and kinase domains of the p110α subunit, which disrupt the autoinhibitory interaction between the catalytic p110α and regulatory p85α subunits. Such structural dissociation enables constitutive activation of PI3Kα independent of upstream extracellular stimuli. Abnormal activation of PI3Kα occurs primarily in *PIK3CA*-mutant breast cancer, endometrial cancer, and various metabolic disorders such as type 2 diabetes [5,6,7].

Current targeted therapeutic strategies against PI3Kα mainly depend on small-molecule specific inhibitors, which regulate pathological signaling pathways through precise structural binding. Most existing PI3Kα inhibitors specifically target and occupy the ATP-binding pocket or allosteric site of the PI3Kα catalytic subunit, thereby effectively blocking the enzymatic phosphorylation activity of PI3Kα. This inhibitory effect reduces excessive PIP_3_ production and the hyperactivation of downstream Akt/mTOR signaling, thereby reversing malignant cellular phenotypes and abnormal metabolic states to achieve therapeutic effects on tumors and metabolic diseases [8,9,10]. In recent years, benefiting from the rapid development of structural biology and computer-aided drug design, the research and development of PI3Kα inhibitors have achieved substantial progress. A large number of structurally optimized novel PI3Kα-selective inhibitors are currently in different stages of preclinical evaluation and phase I-III clinical trials, exhibiting favorable target specificity and promising therapeutic potential. Meanwhile, several potent inhibitors with stable pharmacological properties have completed systematic clinical validation and obtained official marketing approval for clinical application.

Despite the promising therapeutic potential of PI3Kα inhibitors, the clinical application and popularization of approved agents are severely limited by inherent drawbacks. The most prominent challenge is the high incidence of severe systemic adverse reactions and poor patient tolerance during clinical use. Common adverse events include metabolic disorders, gastrointestinal toxicity, and rash [11,12]. These side effects not only impair patients’ quality of life but also frequently lead to dose reduction, treatment interruption, or even drug withdrawal, ultimately compromising overall therapeutic efficacy. Importantly, the most prominent of these adverse events-particularly hyperglycemia, rash, and dermatitis-are generally considered on-target toxicities, because clinically approved PI3Kα inhibitors such as alpelisib inhibit not only mutant but also wild-type p110α. Wild-type PI3Kα signaling is indispensable for the physiological homeostasis of insulin-responsive tissues, including the skin. Allosteric PI3Kα inhibitors that selectively target mutant PI3Kα while sparing the wild-type enzyme are therefore anticipated to substantially reduce these on-target toxicities, representing a major therapeutic advantage of this emerging class. By contrast, off-target adverse effects, which stem from unintended inhibition of additional kinases or PI3K isoforms rather than from the intended PI3Kα target. Non-specific off-target effects and acquired drug resistance after long-term treatment further restrict the long-term clinical efficacy of existing PI3Kα inhibitors. Therefore, the development of high-potency, high-selectivity, low-toxicity, and well-tolerated novel PI3Kα inhibitors, as well as the optimization of individualized medication regimens, has become a core research focus in the field of tumor targeted therapy. Against this research background, this review summarizes PI3Kα and its biological functions in malignancies, analyzes clinical data and structural features of representative PI3Kα inhibitors, adverse reactions, and proposes strategies for their further exploitation and clinical use.

## 2. PI3Kα and PI3Kα/Akt/mTOR Signaling Pathways

### 2.1. PI3Kα

Studies on Class I PI3K advanced considerably during the 1990s, uncovering the structural composition of this kinase, which includes a catalytic subunit (p110) and a regulatory subunit (p85) [13,14]. In 1991, multiple research teams identified via cDNA cloning that p85, which harbors an SH3 domain and two SH2 domains, exhibits no PI3K enzymatic activity and instead functions as an adaptor protein [13]. Subsequently, in 1992, cloning of the p110α cDNA revealed that the p110 subunit itself possesses inherent PI3K activity [13]. PI3Kα, a crucial component of the PI3K family, is classified as a class IA PI3K isoform. The p110α subunit contains five domains: ABD (N-terminal adaptor binding domain), RBD (Ras binding domain), C2, helical, and kinase domains (Figure 1A) [13]. The regulatory subunit p85α also has five domains, including an SH3 domain, a GAP domain, two SH2 domains (nSH2 and cSH2), and an iSH2 domain (Figure 1B) [15]. p85α binds tightly to p110α via iSH2, inhibits its activity at rest, and its nSH2 domain enables PI3Kα to recognize phosphorylated tyrosine residues and restore enzyme activity [16]. The p110α/niSH2 heterodimer resembles a sailboat (Figure S1). Each domain occupies a defined position: ABD (1–108) and RBD (190–291) are linked by a long linker (residues 109–189); C2 (330–480) interacts with iSH2 and is linked to the RBD and helical domain via two distinct linkers corresponding to residues 292–329 and 481–524, respectively; the helical domain (525–696) connects C2 and the kinase domain (697–1068); the kinase domain forms a bilobed catalytic core divided into N- (697–851) and C-terminal (852–1068) regions [13]. The research progress of PI3Kα and the important development nodes of its inhibitors are systematically presented in Figure 2.

### 2.2. PI3Kα/Akt/mTOR Signaling Pathways

The PI3K/Akt/mTOR signaling pathway is the most canonical pathway mediated by the PI3Kα target that is closely associated with tumorigenesis and cancer progression, and it is involved in physiological processes including cell growth, survival, and angiogenesis [17,18,19]. Upon receiving an upstream activation signal, PI3Kα phosphorylates PIP2 to PIP3, further regulating downstream signaling pathways. The binding of PIP3 to the PH domain of Akt leads to a conformational change in Akt, recruited onto the cell membrane and increasing the likelihood of phosphorylation of Thr308 and Ser473 (Figure 3). PDK1 and mTORC2 are key proteins involved in phosphorylating Thr308 and Ser473 (Figure 3). After complete activation, Akt leaves the cell membrane and enters the cytoplasm or nucleus, activating mTORC1 through phosphorylation of two negative regulatory factors (TSC2 and PRAS40), further phosphorylating p70S6K and 4EBP1, regulating cell proliferation and metabolism (Figure 3) [20]. Phosphatase and tensin homolog (PTEN) is a lipid phosphatase that can convert PIP3 to PIP2 through dephosphorylation, thereby exerting a negative feedback regulatory effect on the PI3Kα pathway (Figure 3) [21]. As a typical tumor suppressor, PTEN inactivation leads to activation of the PI3Kα pathway. Within the MAPK/ERK pathway, RAS-GTP in its active state binds and stimulates RAF, which in turn phosphorylates and activates MEK; MEK then drives ERK activation via phosphorylation (Figure 3) [22]. Activated ERK subsequently phosphorylates a diverse array of target proteins located in both the nucleus and cytoplasm. Both the MAPK/ERK and PI3Kα/Akt signaling pathways act as central regulators governing cell survival, proliferation, and growth. Cross-talk between these cascades includes RAS-mediated activation of PI3Kα and ERK-dependent stimulation of mTORC1 (Figure 3) [22,23]. Through such interactions, key factors in the MAPK/ERK signaling cascade can modulate upstream regulators of the PI3K/Akt pathway, ultimately amplifying its intracellular signaling output. Dysfunction of PI3Kα is most common in the occurrence and development of cancer [24].

Excessive PI3Kα/Akt/mTOR cascade activation stems from PI3Kα overactivation, RTK upregulation, as well as PTEN functional deficiency [25]. Aberrant activation of the PI3Kα pathway caused by *PIK3CA* mutations represents the most common oncogenic mechanism in human cancers. Compared with the wild-type protein, *PIK3CA* variants exert gain-of-function effects that drive unregulated cell proliferation [26,27,28]. Mutations are frequently detected in various malignancies, including endometrial cancer [6], breast cancer [29], cervical cancer [30], and head and neck cancer [31]. At the protein structural level, *PIK3CA* mutations are mainly concentrated at three key sites: the helical domain (E542 and E545) and the kinase domain (H1047) [32]. Besides these three hotspot mutations, multiple sporadic mutations have also been identified in PI3Kα. Exon 9 substitutions converting E542 and E545 residues to lysine frequently form electrostatic bonds with the nSH2 domain, disrupting the inhibitory binding between p110α and the p85α regulatory subunit’s N-terminal SH2 region [33]. H1047 is predominantly mutated to arginine (Arg) on exon 20, a frequent alteration in cancers that may enhance p110α activity by interfering with the binding of PIP2 to the lipid membrane [33].

Relevant studies confirmed that inactivation of p110α in model mice can suppress the progression of multiple malignant tumors, especially a variety of solid tumors closely related to abnormal proliferation signals [34]. For tumor microenvironment regulation, the inactivation of p110α can effectively activate CD8+ cytotoxic T cells, promote anti-tumor immune responses, and further induce tumor regression [35,36]. The first highly selective PI3Kα inhibitor, alpelisib was officially approved for clinical application, which confirmed that targeted inhibition of PI3Kα can bring significant survival benefits to tumor patients [37,38].

## 3. Therapeutic Applications of PI3Kα Inhibitors

Owing to the frequent occurrence of *PIK3CA* mutations and the dependence of solid tumors on p110α-mediated signaling, developing inhibitors with high selectivity toward p110α has become a research priority. Accumulating studies demonstrate that PI3Kα is not only essential for tumor-cell growth but also participates in immunomodulatory processes within tumors. Below, we will focus on the application of several PI3Kα inhibitors in tumor treatment.

### 3.1. Application in Breast Cancer

Breast malignancy ranks as the most prevalent cancer type affecting females, with roughly 60% to 70% of cases belonging to the hormone receptor (HR)-positive, human epidermal growth factor receptor 2 (HER2)-negative molecular subtype [39,40]. About 40% of these patients have *PIK3CA* gene mutations, which are one of the core mechanisms leading to tumor occurrence, development, and endocrine therapy resistance. PI3Kα inhibitors have become an important breakthrough in targeted breast cancer therapy, providing precise treatment options for breast cancer patients at different stages and with different drug resistance statuses [41].

ATP-competitive PI3Kα inhibitors are currently the most mature category in clinical application [12]. By simulating the structure of ATP molecules, they competitively bind to the ATP-binding pocket of p110α, directly blocking pathway activation [42]. Alpelisib, as an approved PI3Kα subtype-selective inhibitor, is mainly used for breast cancer, especially for patients with endocrine therapy resistance. A trial showed that alpelisib combined with fulvestrant significantly prolonged progression-free survival (PFS) to 11.0 months compared with 5.7 months for fulvestrant monotherapy [43]. However, it has adverse reactions such as hyperglycemia (63.7%) and rash (53.9%) [43]. Severe adverse events (grade 3/4), predominantly hyperglycemia, occurred in 76.0% of patients receiving alpelisib [44]. Inavolisib is used for *PIK3CA*-mutated HR^+^/HER2^−^ metastatic breast cancer. In the INAVO120 trial, the PFS reached 15 months with inavolisib combined with palbociclib and fulvestrant [44].

In pre-clinical breast-cancer therapeutic settings, CYH33 exhibited more potent antitumor activity in an immune-competent context. CYH33 enhanced the infiltration and activation of CD8+ T and CD4+ T cells, while attenuating M2-like macrophages and regulatory CD4+ T cells. An increase in memory T cells was confirmed by the induction of long-term immune memory with CYH33 treatment. CYH33 promoted fatty acid (FA) metabolism in the TME, while FA enhanced the activity of CD8+ T cells in vitro. The combination of CYH33 with the FA synthase (FASN) inhibitor C75 synergistically inhibited tumor growth with enhanced host immunity [45].

Although allosteric PI3Kα inhibitors have not yet been approved, they have become a research hotspot owing to their high mutation selectivity and reduced toxicity, mainly addressing the resistance problem of ATP-competitive inhibitors [46]. This advantage rests on a mechanistic distinction: ATP-competitive agents inhibit both mutant and wild-type p110α, and blockade of wild-type signaling in insulin-responsive tissues and the skin leads to the high rates of hyperglycemia and rash observed in clinical trials. In contrast, allosteric inhibitors preferentially engage the mutant enzyme while sparing wild-type p110α, which markedly reduces these on-target toxicities. RLY-2608, a pan-mutation-selective allosteric inhibitor, was evaluated in combination with fulvestrant in *PIK3CA*-mutated breast cancer patients who had progressed on CDK4/6 inhibitor therapy and the objective response rate reached 38.7%, while adverse events such as hyperglycemia occurred at a relatively low frequency [46].

Biomarker testing is prerequisite for administering PI3Kα-targeted therapies in HR^+^/HER2^−^ advanced breast cancer. The phase III SOLAR-1 trial established *PIK3CA* mutation as a predictive biomarker for alpelisib plus fulvestrant in HR^+^/HER2^−^ advanced breast cancer [47]. Current biomarker guidelines support *PIK3CA* testing using next-generation sequencing (NGS) of either tumor tissue or plasma circulating tumor DNA (ctDNA) [48]. The ctDNA liquid biopsy provides a less-invasive alternative. The diagnostic performance of ctDNA is strongly affected by tumor fraction: satisfactory sensitivity can be achieved when ctDNA tumor fraction ≥ 2%, but negative liquid-biopsy results should be confirmed with tumor tissue if available, to rule out false-negative findings [48]. The companion-diagnostic landscape is also becoming drug-specific: approved assays are available for *PIK3CA*-directed alpelisib and inavolisib therapy, whereas eligibility for capivasertib encompasses a broader molecular spectrum involving *PIK3CA*, *AKT_1_*, and *PTEN* alterations [48,49,50]. These considerations highlight the increasing importance of comprehensive molecular profiling when selecting among pathway-directed therapies.

### 3.2. Application in Endometrial Cancer

Endometrial carcinoma (EC) ranks among the three most prevalent malignancies of the female genital tract. It can be categorized into two subtypes: estrogen-dependent Type I, which is predominantly endometrioid adenocarcinoma, and estrogen-independent Type II, including serous carcinoma and clear cell carcinoma [51]. Type I patients have a good prognosis, but late-stage, recurrent, and type II EC have limited sensitivity to traditional chemotherapy and endocrine therapy. New targeted drugs are urgently needed [52].

Abnormal activation of PI3K/Akt/mTOR is the most common molecular feature of EC. Overall, *PIK3CA* mutation frequencies in EC range from 12–39% by Sanger sequencing and up to 50% via NGS, with disparities driven by sequencing methodologies. This rate is considerably higher than those of other PI3K subtypes, rendering PI3Kα the primary druggable subtype [53]. Compared with pan-PI3K inhibitors, a PI3Kα-specific inhibitor has lower off-target effects and less toxicity. It has shown good efficacy in clinical research of EC in recent years. The *PIK3CA* hotspot mutations in EC are concentrated in the spiral region (E542K, E545K) and kinase region (H1047R). Mutated PI3Kα continuously activates downstream pathways, leading to tumor cell resistance to chemotherapy and endocrine therapy, and promoting recurrence and metastasis [54].

Alpelisib can significantly inhibit Akt phosphorylation, reduce Cyclin D1 expression, and induce apoptosis in EC with *PIK3CA* mutations/*PTEN* deletions. A study (NCT01219699) aimed to investigate the preliminary efficacy of alpelisib. Antitumor activity was observed when alpelisib was given at doses of 270 mg or above once daily. The overall response rate reached 6.0% [55].

### 3.3. Application in Ovarian Cancer

PI3Kα exerts vital functions in ovarian cancer. When PI3Kα/Akt is activated, it suppresses apoptotic processes while stimulating cell proliferation. Studies have shown that *PIK3CA* mutation or gene amplification is present in 30% of all ovarian cancer cases [56]. These *PIK3CA* mutations enhance the activity of PI3Kα, thereby facilitating improved cell survival, increased cell motility, and accelerated cell cycle progression.

Preclinical studies have shown that PI3Kα inhibitors can enhance the sensitivity of PARP inhibitors (such as olaparib) by down-regulating BRCA1/2 expression and inducing homologous recombination repair (HRR) deficiency, so the combination of the two is considered a potentially effective strategy for ovarian cancer treatment [57]. A phase I clinical trial (dose escalation and expansion cohort) enrolled 28 ovarian cancer patients, using alpelisib combined with olaparib treatment, and determined the recommended phase II dose as alpelisib 200 mg once daily + olaparib 200 mg twice daily. 35.7% of patients achieved partial response (PR) and 50% of patients achieved stable disease (SD). The overall disease control effect was better than monotherapy [57].

### 3.4. Application in Head and Neck Squamous Cell Carcinoma

As the sixth most prevalent malignancy, head and neck squamous cell carcinoma (HNSCC) leads to roughly 800,000 new cases and 400,000 mortalities per year worldwide [58]. The locations of HNSCC include the larynx, nasopharynx, oropharynx, oral cavity, nasal cavity, and sinuses. More than 60% of HNSCC patients are initially diagnosed in the late stage [59]. The PI3K/Akt/mTOR signaling axis undergoes frequent cancer-causing mutations in HNSCC cell line models [60].

A series of clinical trials demonstrated that alpelisib exerts therapeutic benefits in HNSCC patients harboring *PIK3CA* mutations (NCT02282371, NCT03292250, NCT03601507, NCT02145312). CYH33, an oral agent with potent selectivity toward PI3Kα, achieves a 14.3% objective response rate when applied to treat solid malignancies bearing *PIK3CA* mutations such as HNSCC (NCT03544905) [61]. The PI3Kα inhibitory activity of STX-478 against the H1047R site mutation is stronger than that of wild-type and E545K mutant PI3Kα. STX-478 demonstrated robust efficacy in human tumor xenografts without causing the metabolic dysfunction observed with alpelisib [62].

### 3.5. Application in Cervical Cancer

Human papillomavirus (HPV) infection is recognized as the primary driver of cervical cancer development. Through the combination of relevant research analyses, along with functional experiments conducted on cervical cancer cell lines and immune-related detection assays, three distinct subtypes of cervical cancer have been classified: (I) *PIK3CA* wild-type without YAP1 amplification, (II) *PIK3CA*-mutant without YAP1 amplification, and (III) *PIK3CA* wild-type with YAP1 amplification. YAP1 amplification is closely correlated with inferior survival outcomes in patients with cervical cancer [63].

Alpelisib and inavolisib, two inhibitors with high specificity for PI3Kα, have been shown to selectively suppress the proliferation of various cervical cancer cell lines harboring *PIK3CA* mutations, while exerting little to no inhibitory impact on cells with wild-type *PIK3CA*. Additionally, the combination of alpelisib and T-cell therapy was found to enhance the tumor-killing ability of T cells, with the most significant therapeutic effects achieved when the drug was administered as a pretreatment before T-cell exposure. Furthermore, the combination of PI3K inhibition and antigen-specific immunotherapy has emerged as a promising therapeutic approach to improve treatment outcomes in patients with advanced cervical cancer associated with HPV16 [63].

In addition to the well-documented efficacy of PI3Kα inhibitors in the previously mentioned tumors, growing evidence supports their utility in treating other malignancies such as lung [64], colorectal [65], and prostate cancers [66]. Looking ahead, continued investigation is poised to uncover an expanded range of clinical indications for these selective PI3Kα inhibitors.

## 4. PI3Kα Inhibitors and Competing AKT-Targeted Therapies

To date, multiple selective PI3Kα inhibitors have been successfully developed for tumor-targeted therapy. Among them, alpelisib and inavolisib have been approved by the FDA, while CYH33 has obtained marketing approval from the Japanese Ministry of Health, Labour and Welfare. Additionally, several highly selective PI3Kα inhibitors, including serabelisib, taselisib, CH5132799, STX-478, and RLY-2608, are currently in different stages of clinical research, and most candidate compounds are still limited to the preclinical stage. The structures of the representative PI3Kα inhibitors are shown in Figure 4.

### 4.1. ATP-Competitive PI3Kα Inhibitor

Developed by Novartis, alpelisib represents the first oral PI3Kα-targeting agent granted FDA approval in May 2019 for breast cancer therapy. Its systematic chemical designation is (2*S*)-1-N-[4-methyl-5-[2-(1,1,1-trifluoro-2-methylpropan-2-yl)-4-pyridinyl]-1,3-thiazol-2-yl]pyrrolidine-1,2-dicarboxamide (Figure 4). The combination regimen of alpelisib with fulvestrant has been authorized in the United States for post-menopausal females and males suffering from HR^+^, HER2^−^ advanced or metastatic breast cancer harboring *PIK3CA* mutations [13]. Biochemical tests conducted in vitro have reported that its half-maximal inhibitory concentration (IC_50_) values against p110α, p110β, p110δ and p110γ are 4.6 nM, 1156 nM, 290 nM and 250 nM, respectively (Table 1) [67].

In addition, the alpelisib’s efficacy in treating conditions such as stomach cancer (NCT04526470), meningioma (NCT03631953), colorectal cancer (NCT04753203), endometrioid endometrial cancer (NCT05154487), renal cell cancer and pancreatic tumors (NCT02077933), oropharyngeal cancer (NCT03601507), *PIK3CA*-related overgrowth spectrum (PROS, NCT04085653), and HNSCC (NCT04997902) is currently in clinical trials. Table 2 summarizes comprehensive data regarding relevant clinical trials.

As an oral novel PI3Kα inhibitor developed by Genentech, inavolisib (Itovebi) received FDA approval in October 2024 based on the INAVO120 trial, which evaluated a triplet combination of inavolisib (selective PI3Kα inhibitor with a dual mechanism of kinase inhibition and mutant p110α degradation) plus palbociclib plus fulvestrant [68]. This regimen is indicated for adults with *PIK3CA*-mutated, HR^+^/HER2^−^ locally advanced or metastatic breast cancer after recurrence on or after adjuvant endocrine therapy. Its systematic chemical name is (2*S*)-2-[[2-[(4*S*)-4-(difluoromethyl)-2-oxo-1,3-oxazolidin-3-yl]-5,6-dihydroimidazo [1,2-*d*][1,4]benzoxazepin-9-yl]amino]propanamide (Figure 4). In cell-free biochemical analyses, the IC_50_ values of inavolisib against p110α, β, δ and γ were 0.034, 100, 12 and 18 nM, respectively (Table 1) [13]. In the INAVO120 trial, this triplet regimen achieved a median PFS of 15.0 months at primary analysis versus 7.3 months with placebo plus palbociclib plus fulvestrant (HR 0.43; *p* < 0.0001), and updated data at 34.2 months of median follow-up showed a sustained benefit with median PFS of 17.2 months [68].

In addition, ongoing clinical trials are currently evaluating the therapeutic efficacy of inavolisib for the treatment of multiple malignant diseases, including endometrial cancer (NCT07522697), prostate cancer (NCT07287150), and colorectal cancer (NCT07323576). Table 3 summarizes comprehensive data regarding relevant clinical trials.

CYH33 (Risovalisib), developed by Shanghai Hahe Biopharma, is an orally active and highly selective PI3Kα inhibitor approved in Japan in March 2026. Its chemical name is N-[5-[6-[(4-methylsulfonylpiperazin-1-yl)methyl]-4-morpholin-4-ylpyrrolo [2,1-f][1,2,4]triazin-2-yl]-4-(trifluoromethyl)-2-pyridinyl]carbamate (Figure 4). Recently, CYH33 has been authorized as a single agent in Japan for patients with *PIK3CA*-mutated advanced or recurrent ovarian clear cell carcinoma progressing after chemotherapy. In an in vitro biochemical assay, the IC_50_ values for p110α, β, δ, and γ were 5.9, 600, 79, and 225 nM, respectively, demonstrating high selectivity toward the α isoform (Table 1) [13]. Beyond its direct antitumor effects, CYH33 exerts immunomodulatory influences on the tumor microenvironment: it upregulates the activity of CD8+ and CD4+ T cells, while simultaneously suppressing the function of M2 macrophages and regulatory T cells [45]. Furthermore, ongoing clinical trials are evaluating the therapeutic effects of the agent in patients with breast cancer (NCT04856371) and PROS (NCT06975618). Table 4 summarizes comprehensive data regarding relevant clinical trials.

Serabelisib, developed by Takeda, is an orally available, highly selective PI3Kα inhibitor currently under phase II clinical investigation for solid tumors. Its chemical name is [6-(2-amino-1,3-benzoxazol-5-yl)imidazo [1,2-a]pyridin-3-yl]-morpholin-4-ylmethanone (Figure 4). To date, serabelisib is being evaluated both as monotherapy and in combination regimens for advanced or metastatic solid malignancies harboring *PIK3CA* mutations, including breast cancer, EC, and non-small-cell lung cancer, in multiple phase I/II trials. In an in vitro biochemical assay, the IC_50_ values for p110α, β, δ and γ were 15, 4500, 13,900 and 1900 nM, respectively, demonstrating >100-fold selectivity for PI3Kα over other class I PI3K isoforms (Table 1) [69]. In addition, clinical trials are also evaluating the efficacy of this drug for gastric or gastroesophageal adenocarcinoma (NCT02551055) and clear-cell metastatic renal cell carcinoma (NCT02724020). Table 5 summarizes comprehensive data regarding relevant clinical trials.

Taselisib, discovered by Genentech, is an orally bioavailable, potent class I PI3K inhibitor with preferential activity against the α, δ, and γ isoforms, and reduced potency toward p110β. Its chemical name is 2-methyl-2-[4-[2-(5-methyl-2-propan-2-yl-1,2,4-triazol-3-yl)-5,6-dihydroimidazo [1,2-*d*][1,4]benzoxazepin-9-yl]pyrazol-1-yl]propanamide (Figure 4). Although initially investigated for HR^+^/HER2^−^, *PIK3CA*-mutated advanced or metastatic breast cancer, taselisib’s clinical development was discontinued by Roche in June 2018 after the phase III SANDPIPER trial showed only modest efficacy but excessive toxicity, particularly gastrointestinal adverse events. In an in vitro biochemical assay, the IC_50_ (or K_i_) values for p110α, β, δ, and γ were 0.29, 9.1, 0.12, and 0.97 nM, respectively, indicating high potency against the α isoform while sparing β (Table 1) [70]. Several clinical studies have investigated taselisib in combination with enzalutamide for metastatic breast cancer (NCT02457910, terminated at interim analysis due to toxicity), and as a single agent for advanced lymphoma, refractory multiple myeloma and other malignancies (NCT04439175, active, not recruiting). Table 6 summarizes comprehensive data regarding relevant clinical trials.

### 4.2. Allosteric PI3Kα Inhibitors

RLY-2608 has a stronger inhibitory effect on the activity of PI3Kα mutant (H1047R, IC_50_: 4 nM) than the wild type (IC_50_: 48 nM) (Table 7). In preclinical tumor xenograft models, RLY-2608 achieved potent antitumor activity without significantly altering insulin levels, thereby dissociating its efficacy from the metabolic side effects seen with earlier agents [71]. The Phase I ReDiscover trial validated sustained target suppression in individuals with *PIK3CA*-altered solid tumors, accompanied by mild effects on glucose metabolism, manageable safety, and potent antitumor activity [71]. In addition, clinical trials are also evaluating the efficacy of this drug for PROS and MCAP (NCT06975618) (Table 8).

STX-478, also named tersolisib, was originally developed by Scorpion Therapeutics and acquired by Eli Lilly in January 2025. It is an orally administered mutant-selective allosteric PI3Kα inhibitor currently under phase I/II clinical research for *PIK3CA*-mutated advanced solid tumors. Its chemical structure enables preferential binding to mutant PI3Kα rather than the wild-type isoform, effectively reducing typical metabolic toxicities such as hyperglycemia. In vitro assays confirm it exerts a potent inhibitory effect on common hotspot mutants including H1047R, E542K, and E545K. In an in vitro biochemical assay, the IC_50_ values for WT PI3Kα and the variants H1047R, E542K, and E545K were 131, 9.4, 113, and 71 nM, respectively (Table 7) [62]. STX-478 exerts potent antitumor effects in human tumor xenograft models, while avoiding metabolic side effects commonly induced by alpelisib. Its combination with fulvestrant or CDK4/6 inhibitors shows satisfactory safety tolerance and achieves stable and sustained tumor suppression in ER^+^ and HER2^−^ tumor xenografts [62].

LOXO-783 is a brain-penetrant allosteric inhibitor with over 50-fold selectivity for H1047R. In breast cancer models, it induced tumor regression as a single agent without causing hyperglycemia or elevating plasma insulin levels. However, modest efficacy (3% ORR) in the Phase 1 trial (NCT05307705) led to the discontinuation of its clinical development (Table 8). OKI-219 and CGT4824 achieved high selectivity for the H1047R mutation (approximately 100-fold and 15-fold, respectively). Both drugs have shown activity against intracranial tumors in preclinical models, and OKI-219 is currently being studied in the phase I PIKture-01 trial (NCT06239467) for H1047R mutant advanced solid tumor patients [9].

### 4.3. Competing Akt-Targeted Therapies

Beyond PI3Kα-selective kinase inhibitors, the Akt inhibitor capivasertib represents an important therapeutic option for HR^+^/HER2^−^ metastatic breast cancer patients with Akt-pathway aberrations including *PIK3CA* activating mutations [72]. In the phase III CAPItello-291 trial, capivasertib combined with fulvestrant significantly improved PFS versus placebo plus fulvestrant in patients previously progressed on aromatase-inhibitor-based therapy, with the most pronounced clinical benefit observed in tumors harboring *PIK3CA*, *AKT_1_*, or *PTEN* alterations (median PFS 7.3 months vs. 3.1 months) [72]. This combination has received US-FDA approval and is incorporated into updated ASCO clinical practice recommendations for second- and later-line treatment, as an alternative regimen alongside alpelisib-fulvestrant for *PIK3CA*-altered HR^+^/HER2^−^ metastatic breast cancer [72]. Distinct from alpelisib, which targets PI3Kα itself, capivasertib acts downstream of PI3Kα; accordingly, it retains efficacy in settings where PI3Kα inhibitors may fail, although it is also associated with characteristic toxicities including rash, diarrhoea and hyperglycaemia, which can lead to treatment discontinuation [72]. Notably, capivasertib provides therapeutic benefit not only for *PIK3CA*-mutant tumors but also for tumors driven by *AKT_1_* mutation or *PTEN* loss, expanding its biomarker-defined patient population compared with PI3Kα-specific inhibitors [72].

## 5. Toxicity of PI3Kα Inhibitors

Toxicities of PI3Kα inhibitors fall into two categories: on-target and off-target. On-target toxicities result from inhibition of the intended enzyme in normal tissues. Approved agents such as alpelisib inhibit both mutant and wild-type p110α. Wild-type PI3Kα signaling is essential for the physiological homeostasis of insulin-responsive tissues, including the skin. Therefore, blocking the wild-type enzyme causes toxicities, mainly hyperglycemia and skin reactions. These effects are inherent to the mechanism of orthosteric PI3Kα inhibitors. Allosteric inhibitors that target only mutant PI3Kα and preserve wild-type signaling may greatly reduce these toxicities, which is a major clinical advantage. Off-target toxicities occur when a drug acts on proteins or pathways other than its intended target. For PI3Kα inhibitors, off-target effects mainly involve inhibition of other class I PI3K isoforms or engagement of unrelated kinases. For example, PI3Kδ inhibition causes immunomodulatory toxicities such as pneumonitis, colitis, and hepatotoxicity.

### 5.1. Hyperglycemia

Mounting evidence confirms that the p110α subunit of PI3K dominates the cellular transduction of insulin signals under diverse disease states, most notably cancers. Small-molecule inhibitors selectively targeting p110α interrupt insulin-dependent signaling pathways; this blockade facilitates glycogen catabolism within the liver and impedes glucose uptake by skeletal muscle and adipose depots, consequently leading to hyperglycemic complications [11]. Signs of hyperglycemia involve polydipsia, polyuria, polyphagia, and unintentional weight loss. If diabetic ketoacidosis occurs sporadically, initial saline hydration is required, combined with insulin and potassium replacement; bicarbonate or phosphate supplements are given for persistent acidosis or severe phosphate depletion [73]. Hyperglycemia is considered an on-target effect of PI3Kα inhibition [74].

In the phase III SOLAR-1 trial of alpelisib (Piqray), the first globally launched selective PI3Kα inhibitor, 6.3% of patients discontinued treatment from any-grade hyperglycemia, while 4.2% withdrew therapy owing to grade 3 or worse hyperglycemia [74]. All-grade hyperglycemia occurred in 65% of alpelisib recipients; severe (grade ≥ 3) hyperglycemia was seen in 37% [74]. In the INAVO120 study, 96% (52/54) of patients with fasting blood glucose(FBG) > 160 mg/dL improved by ≥1 grade with inavolisib; median improvement onset took 8 days [73].

For both alpelisib and inavolisib, hyperglycemia mostly arises during the first month of treatment. Frequent rigorous FBG surveillance helps early diagnose hyperglycemia, with samples collected pre-dose after an 8–12 h overnight fast. Alpelisib requires weekly FBG checks in the first two weeks and monthly follow-ups afterward, plus quarterly HbA1c testing; SOLAR-1 added a Day 8 visit via protocol revision for hyperglycemia screening [73]. Per US inavolisib labeling, FBG and HbA1c should be assessed and glucose optimized before treatment and monitored regularly long-term [73].

Moreover, patients with alpelisib-related hyperglycemia frequently receive anti-hyperglycemic therapy, and metformin is the most widely used agent [75]. With accumulating clinical data, it has been noted that more than half of patients receiving alpelisib require additional glucose-lowering agents besides metformin. The therapeutic effect of metformin is partially counteracted by PI3K inhibition, and overlapping gastrointestinal toxicities may restrict its dose escalation [75]. SGLT2 inhibitors are a newer class of diabetic medications that inhibit renal glucose reabsorption. In preclinical studies, SGLT2 inhibitors successfully suppressed PI3K inhibitor-induced hyperglycemia [75]. Clinically, several case reports have highlighted success in managing alpelisib-associated hyperglycemia with SGLT2 inhibitors; use of these agents is associated with greater reductions in serum glucose levels compared with metformin. Dietary strategies may also be successful adjuncts to reducing hyperglycemia and may even contribute anti-tumor effects [75].

### 5.2. Skin-Related Adverse Reactions

Skin-related adverse reactions, predominantly cutaneous rash, represent a common pharmacological on-target side effect of PI3Kα inhibition [74]. Maculopapular rash, itching, and xerosis rank among the most prevalent cutaneous complications triggered by PI3K inhibition. Histamine-secreting cells alongside eosinophils contribute to the emergence of drug-induced rash from PI3K inhibitors [74]. Among subjects receiving alpelisib for two weeks, those presenting with skin rash exhibited markedly elevated peripheral blood eosinophil ratios, implying this parameter could serve as a biomarker for rash onset [74].

Cutaneous rash commonly emerges during the initial month of therapy with alpelisib and inavolisib. Notably, no serious cutaneous adverse events, including Stevens-Johnson syndrome, toxic epidermal necrolysis, or erythema migrans, have been documented among inavolisib-treated individuals [73]. Of the total 102 enrolled subjects, all-grade cutaneous rash occurred in 41 cases (40.2%), and roughly half of these affected individuals (19 patients, 46.3%, accounting for 18.6% of the whole cohort) progressed to Grade 3 severe skin lesions. Antihistamine prophylaxis was administered to 42% (43 out of 102) participants [76]. For 23 sufferers with limited-area skin eruptions, lesions most commonly appeared on the torso, including the chest, belly, and dorsal region (18 patients,78%) as well as limbs (16 patients,70%). Facial rash was observed merely in two patients (9%), whilst scalp involvement was found in a single case (4%). Additionally, erythroderma covering over 80% of total body surface emerged in three patients, equivalent to 13% of this subgroup [76].

All individuals newly prescribed alpelisib are advised to receive preventive antihistamine medication. Non-sedating options including 10 mg cetirizine, 10 mg loratadine or 180 mg fexofenadine can be taken once daily for the initial eight treatment weeks to prevent rash development, followed by gradual dose reduction [74]. If cutaneous side effects emerge at any subsequent timepoint, antihistamine regimens ought to be resumed promptly. Since these medicines deliver limited therapeutic benefits after rash has already developed, their primary clinical value lies in preemptive prevention rather than post-onset intervention [74].

### 5.3. Diarrhea

Hyperglycemia and cutaneous rash tend to arise shortly after alpelisib commencement, whereas diarrheal adverse events develop comparatively later in the treatment course. Among 164 alpelisib-treated participants suffering from diarrheal complications, around 63.4% (104 individuals) were given symptomatic supportive drugs. Antidiarrheal agents dominated these prescriptions, with loperamide being the most commonly utilized option, used in 69 out of the 104 medicated patients (66.3%) [77].

### 5.4. Additional Adverse Events

Stomatitis stands as one prevalent adverse event among the 284 alpelisib recipients, occurring at an incidence of 24.6% according to published data [74]. Once early signs of aphthous oral inflammation emerge, patients are prescribed dexamethasone oral rinse three times daily for four to six weeks via swish-and-spit administration, with further usage restricted to symptom-based requirements only. Triamcinolone-containing corticosteroid dental paste serves as an alternative option; sufferers are told to coat damaged oral mucosa two to four times daily until mucosal injuries fully heal. As documented in the SOLAR-1 trial, nausea (44.7%) and vomiting (27.1%) also frequently develop, which require targeted pharmacological intervention plus regular clinical follow-up [74].

Pneumonitis and pancreatitis are uncommon adverse outcomes in this patient cohort, seen in 0.7% (2 subjects) and 0.4% (1 subject) of participants separately. Conversely, copanlisib and idelalisib are linked to a substantially higher risk of pneumonitis [78]. When interstitial lung inflammation is clinically suspected, high-resolution chest CT scans are required to validate diagnosis and exclude infectious interstitial lung ailments [74]. Clinicians may arrange inpatient care, bronchoalveolar lavage or tissue biopsy where necessary. Specialist consultation with respiratory physicians alongside high-dose steroid regimens is recommended, and antibacterial agents will be introduced upon suspicion of infectious origins [79].

## 6. The Binding Characteristics of PI3Kα Inhibitors and PI3Kα

The current mainstream PI3Kα inhibitors are ATP-competitive inhibitors (alpelisib, inavolisib, and taselisib) and allosteric inhibitors (RLY-2608 and STX-478). ATP competitive inhibitors can occupy the ATP binding pocket and interact with active pocket residues such as Ser774, Lys802, Tyr836, VAL851, Ser854, and GLN859. Within the ATP pocket, alpelisib forms contacts with Val851, Gln859, Ser854, and Lys802 (Figure 5A,B) [13]. Among these, the dual hydrogen bonds formed between alpelisib and the hinge-region residue Gln859 are particularly essential for the isoform selectivity of PI3Kα. Inavolisib engages in a set of highly specific interactions within the PI3Kα ATP pocket: it forms hydrogen bonds with Val851, Gln859, Ser854, and Tyr836, while its CF_2_ group interacts with the hydroxyl moiety of Ser774 (Figure 5C,D) [13]. These precise binding interactions contribute to its enhanced selectivity for the PI3Kα isoform. Taselisib utilizes the same carboxamide as alpelisib and inavolisib to selectively inhibit PI3Kα activity by interacting with Ser854, Gln859, and Val851 (Figure 5E,F) [13]. The difference in their binding affinity is due to ligand-specific interactions, rather than conformational changes in the ATP pocket.

Allosteric PI3Kα inhibitors can target mutation-specific conformations or cryptic sites outside the ATP pocket, achieving higher selectivity for mutant forms, lower off-target toxicity, and reduced risk of drug resistance compared to traditional ATP-competitive inhibitors. In STX-478, the urea group forms a bifurcated hydrogen bond with Leu911 and a suboptimal hydrogen bond with Gly912. Meanwhile, the benzofuran and trifluoromethyl moieties occupy the deepest region of the pocket, anchored by interactions with surrounding hydrophobic and aromatic residues (Figure 6A,B) [33]. RLY-2608 binds the allosteric site via three distinct mechanisms: (1) its aminoisoxindole core fills the pocket effectively, establishing enhanced interactions with Tyr1021 and a more robust hydrogen bond to Asp1018′s backbone; (2) the 5-fluoro phenyl group optimally occupies hydrophobic pockets; (3) the 2-chloro substituent forms a halogen bond with the backbone carbonyl of Glu1012 (Figure 6C,D) [32]. The ligand-specific interactions distinguish their binding modes, and the presence of these interactions stabilizes PI3Kα in an inhibited state. Allosteric PI3Kα inhibitors provide a breakthrough strategy for targeting carcinogenic *PIK3CA* mutations. This type of inhibitor achieves higher selectivity and lower metabolic toxicity compared to traditional ATP competitive inhibitors by utilizing mutation specific conformation and hidden binding sites.

## 7. Challenges and Development Strategies for New PI3Kα Inhibitors

PI3K inhibitor development continues to face significant challenges, foremost among them the problem of drug resistance. The therapeutic effect of PI3Kα inhibitors is restricted by intrinsic and acquired resistance. Functional inactivation or genomic loss of PTEN constitutes a key molecular driver of acquired resistance to PI3Kα inhibitor therapy. Following exposure to alpelisib, metastatic sites may develop diverse secondary *PTEN* alterations (such as copy number deletion, frameshift indels, and splice site mutations), leading to convergent loss of PTEN protein function [80]. Subsequent analyses of patients treated with alpelisib plus aromatase inhibitors similarly identified PTEN loss-of-function alterations in resistant tumors and demonstrated the expansion of activating *ESR1* mutations in number and allele fraction during treatment, which were associated with resistance [81]. Studies found that PI3K antagonists inhibited Akt and relieved the repression of receptor tyrosine kinase expression and activity. Such feedback relief impeded sustained PI3K/Akt pathway inhibition and attenuated responses to these agents [82]. Studies revealed that growth factors such as insulin-like growth factor 1 and neuregulin 1 are capable of activating mTOR and mediating resistance to alpelisib. These findings suggest that concomitant administration of mTORC1 inhibitors may enhance the clinical activity of p110α-targeted agents and delay the emergence of resistance [83]. Collectively, these observations indicate that resistance to PI3Kα inhibitors is not mediated by a single genomic event but instead reflects dynamic pathway rewiring involving PTEN loss, endocrine-resistance alterations, receptor tyrosine kinase feedback, and reactivation of downstream mTORC1 signaling.

Off-target effects are also a major obstacle to clinical translation. The high structural homology among ATP-binding pockets gives rise to adverse reactions. As a classic PI3Kα inhibitor, alpelisib suffers from serious dose-related toxicities. Similar to earlier candidates, it acts as an ATP-competitive agent. Elevated drug levels will inevitably cause inhibition of other PI3K isoforms. PIP2 lipids bind to the cell membrane with low affinity, which creates a shallow substrate-binding pocket and prevents inhibitors from maintaining stable binding for a prolonged period. Moreover, the protein surface is surrounded by the membrane, which further impedes the access of small molecules. Small-molecule inhibitors have to be used at higher concentrations to target the hinge region, thereby triggering adverse reactions from non-specific binding.

Targeted anticancer drugs can exert immunomodulatory effects, which provides a strong rationale for developing novel candidates for combination with immunotherapy to improve clinical outcomes [84]. BEBT-908 is a PI3Kα/HDAC inhibitor that can effectively inhibit tumor cell growth. BEBT-908 can activate the host’s anti-tumor immune response and enhance immune checkpoint blockade therapy [84]. It is a promising targeted therapy for multiple types of cancer that can enhance the efficacy of immunotherapy.

Proteolysis-targeting chimeras (PROTACs) represent another frontier. These bifunctional molecules recruit E3 ubiquitin ligases to degrade target proteins [85]. ZM-PI05 is a new PI3K-p110α PROTAC, which can effectively inhibit the proliferation of breast cancer cells by degrading PI3K-p110α. It can significantly reduce cell proliferation and induce G1-phase cell cycle arrest [86]. PI3Kα-targeting degrader-antibody conjugates (DACs), a targeted drug delivery strategy, are currently under preclinical investigation. As reported in a 2025 AACR-NCI-EORTC conference poster, AC4847-based DACs can induce antigen-dependent PI3Kα degradation, exert potent anti-tumor activity, and avoid the hyperglycemia and insulin resistance associated with systemic wild-type PI3Kα inhibition [87].

Covalent inhibitors utilize reactive electrophilic groups to form irreversible linkages with non-conserved cysteine residues, thereby enhancing both target selectivity and the duration of therapeutic effect. CNX-1351 is a covalent inhibitor targeting PI3Kα, which can covalently bind to Cys862, an amino acid unique to the α subtype. This covalent modification does not occur in PI3K β/γ/ δ [88]. CNX-1351 is easily metabolized rapidly in rat liver microsomes. Compound 19 is a potent covalent PI3Kα inhibitor and exhibits excellent stability in rat liver microsomes, demonstrating long-term inhibitory effects on PI3Kα [89]. Covalent inhibitors have opened up important directions for the development of new-generation PI3Kα-targeted drugs.

The targeted drug delivery strategy for tumors is also an effective measure to reduce the toxic side effects of PI3Kα inhibitors and enhance therapeutic efficacy. The typical PI3K inhibitor LY294002 embodies this challenge, as its poor solubility and short half-life hinder its clinical development [90]. A key solution is SF1126, an RGDS-labeled LY294002 prodrug. This modification aims to enhance solubility and achieve targeted delivery by binding to integrins overexpressed in tumors, thereby directing active drugs to the tumor vascular system and parenchyma [13]. This approach can effectively improve the biocompatibility of drugs, stability in the blood, tumor targeting, tendency to remain in tumor tissue, and internalization by tumor cells.

The optimal sequencing of pathway-directed and endocrine therapies after progression on a CDK4/6 inhibitor remains an important unresolved clinical question, especially for patients with breast cancer. Rather than supporting a universal treatment sequence, current evidence favors a biomarker-driven approach. For patients with *PIK3CA*-mutated tumors, alpelisib plus fulvestrant is an established option based on SOLAR-1 and subsequent clinical experience [47]. Capivasertib plus fulvestrant provides an alternative pathway-directed strategy for tumors harboring *PIK3CA*, *AKT_1_*, or *PTEN* alterations; notably, a large proportion of patients enrolled (69.1%) in CAPItello-291 had previously received a CDK4/6 inhibitor, supporting its relevance in the contemporary post-CDK4/6 setting [91]. In patients with acquired *ESR1* mutations, the oral selective estrogen receptor degrader (SERD) elacestrant represents another biomarker-directed option following prior endocrine therapy and CDK4/6 inhibition, as demonstrated in the phase III EMERALD trial [92]. Other therapeutic alternatives consist of endocrine-therapy-based regimens incorporating the mTOR inhibitor everolimus, which may be administered regardless of tumor genomic profiles [72]. Therefore, selection among PI3Kα inhibition, Akt inhibition, mTOR inhibition, and next-generation endocrine therapy should incorporate *PIK3CA/AKT_1_/PTEN* and *ESR1* status, previous endocrine sensitivity, prior exposure to targeted agents, disease burden, and toxicity risks [48,72].

## 8. Discussion

In the past few decades, although significant progress has been made in the development of PI3Kα inhibitors, clinical translation is still hindered by dose-limiting toxicity and off-target effects. Common adverse events, such as high blood sugar, rash, and diarrhea, often force dose adjustments or discontinuation, thereby limiting efficacy. Although combination therapy can improve outcomes and potentially reduce individual drug-related toxicity, treatment tolerance remains an unresolved issue that requires further optimization. The advances in structural biology (cryo-electron microscopy, X-ray crystallography, molecular simulations) have elucidated the dynamics of PI3K α, making it possible to design next-generation inhibitors rationally. Mutants targeting non-ATP sites exhibit higher selectivity and lower toxicity, while emerging strategies such as mutation-selective degradation agents, PROTACs, and covalent inhibitors address traditional limitations by enhancing mutation specificity and overcoming resistance. Future research will reveal the detailed role of PI3K alpha in cancer and other diseases, develop more specific small molecules, and translate new therapies into clinical applications.

## Figures and Tables

**Figure 1:**
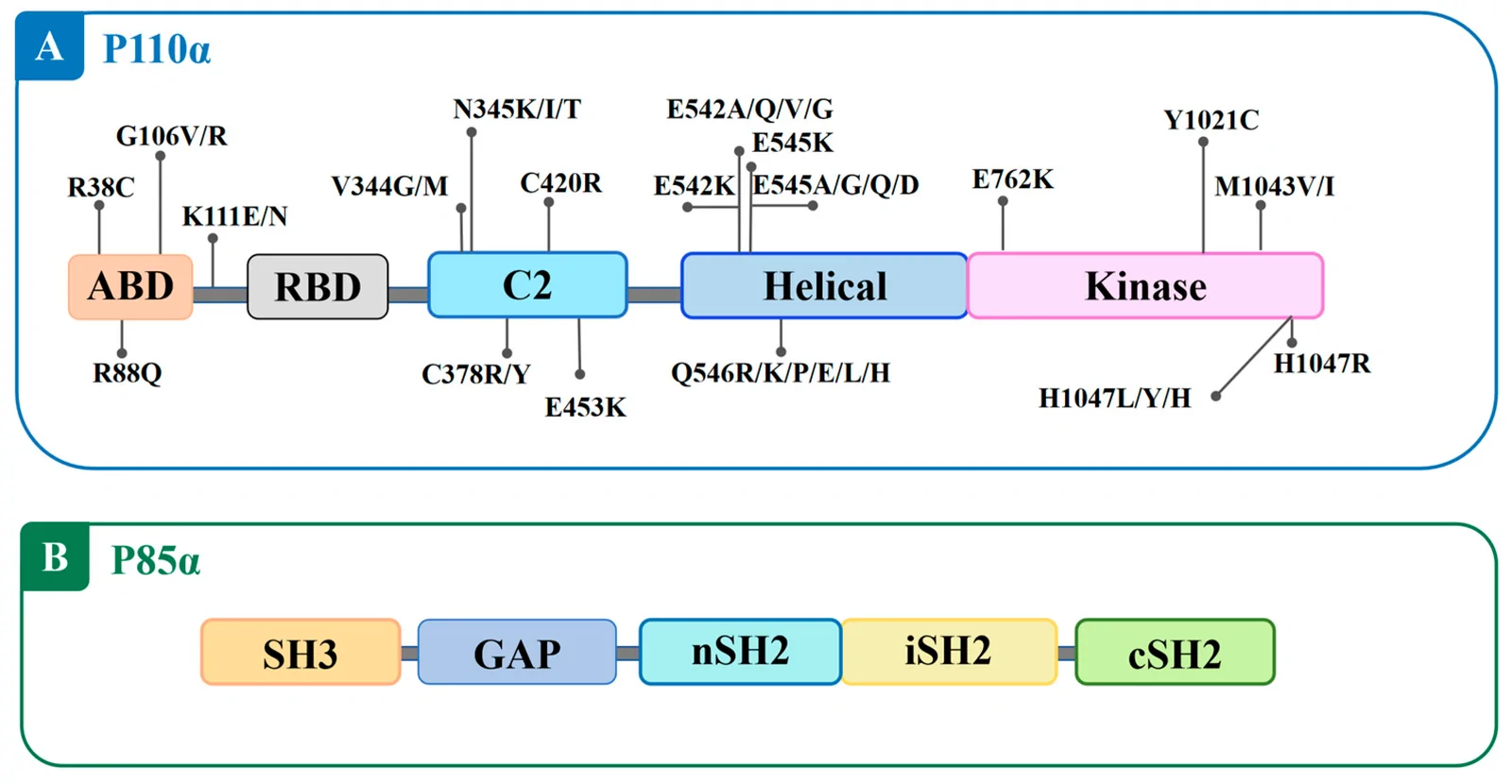
(**A**) p110α sequence and frequent oncogenic mutations in p110α. (**B**) P85α sequence.

**Figure 2:**
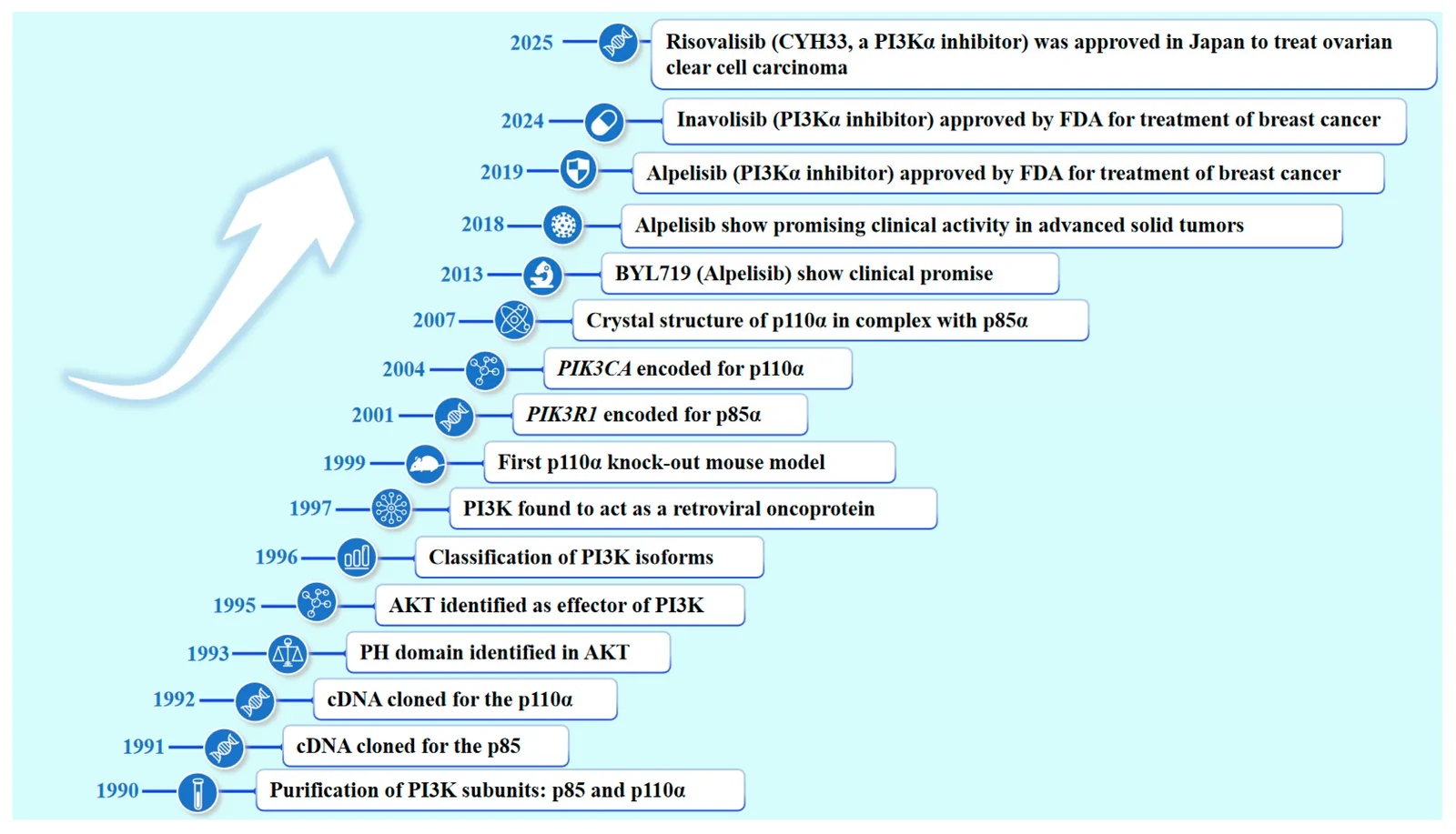
A brief history of PI3Kα and PI3Kα inhibitors.

**Figure 3:**
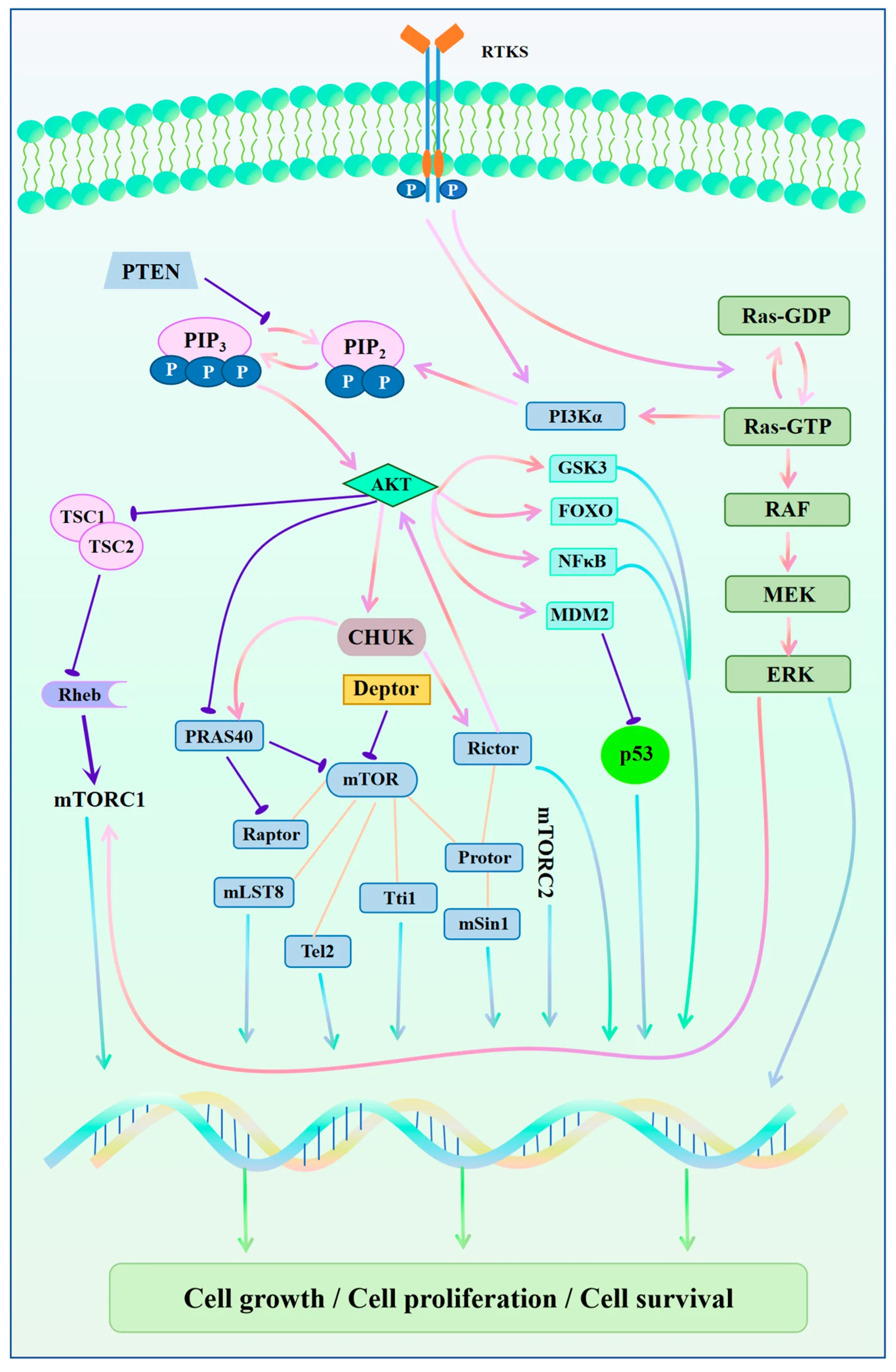
PI3Kα/Akt/mTOR signaling pathways. Upon activation, RTKs can stimulate PI3Kα, which catalyzes the conversion of PIP_2_ to PIP_3_, whereas PTEN negatively regulates this reaction in a reverse manner. Once Akt is activated by PIP_3_, it promotes mTOR signaling (existing as two distinct complexes, mTORC1 and mTORC2) by suppressing negative regulators including TSC1/2. Meanwhile, Akt also modulates a series of downstream effectors such as GSK3, FOXO, NFκB, and MDM2. The MAPK/ERK cascade can target upstream regulators of the PI3K/Akt axis, including PI3Kα and mTORC1, thereby strengthening its signaling intensity. These signaling cascades are involved in multiple key biological processes.

**Figure 4:**
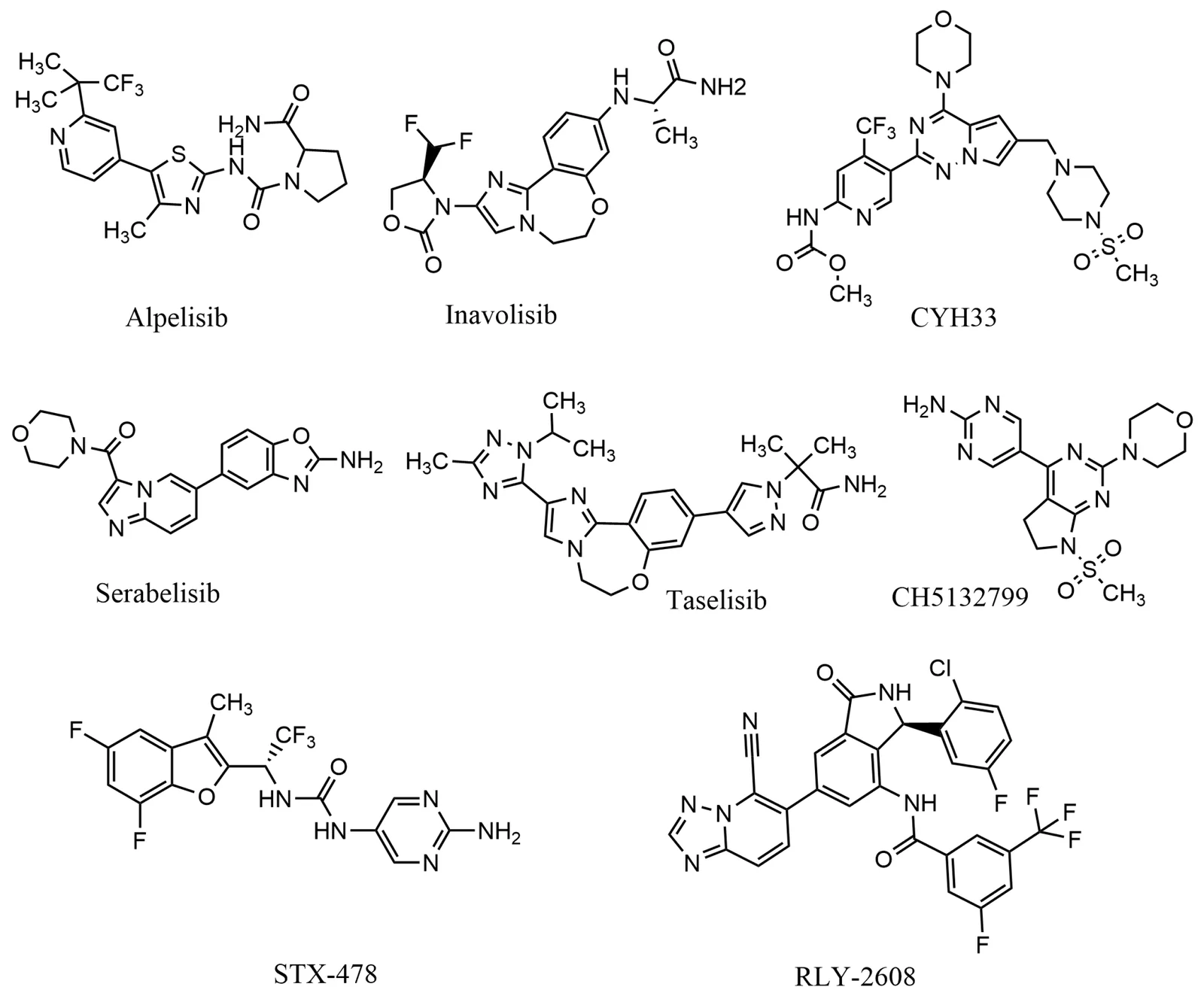
Representative PI3Kα inhibitors.

**Figure 5:**
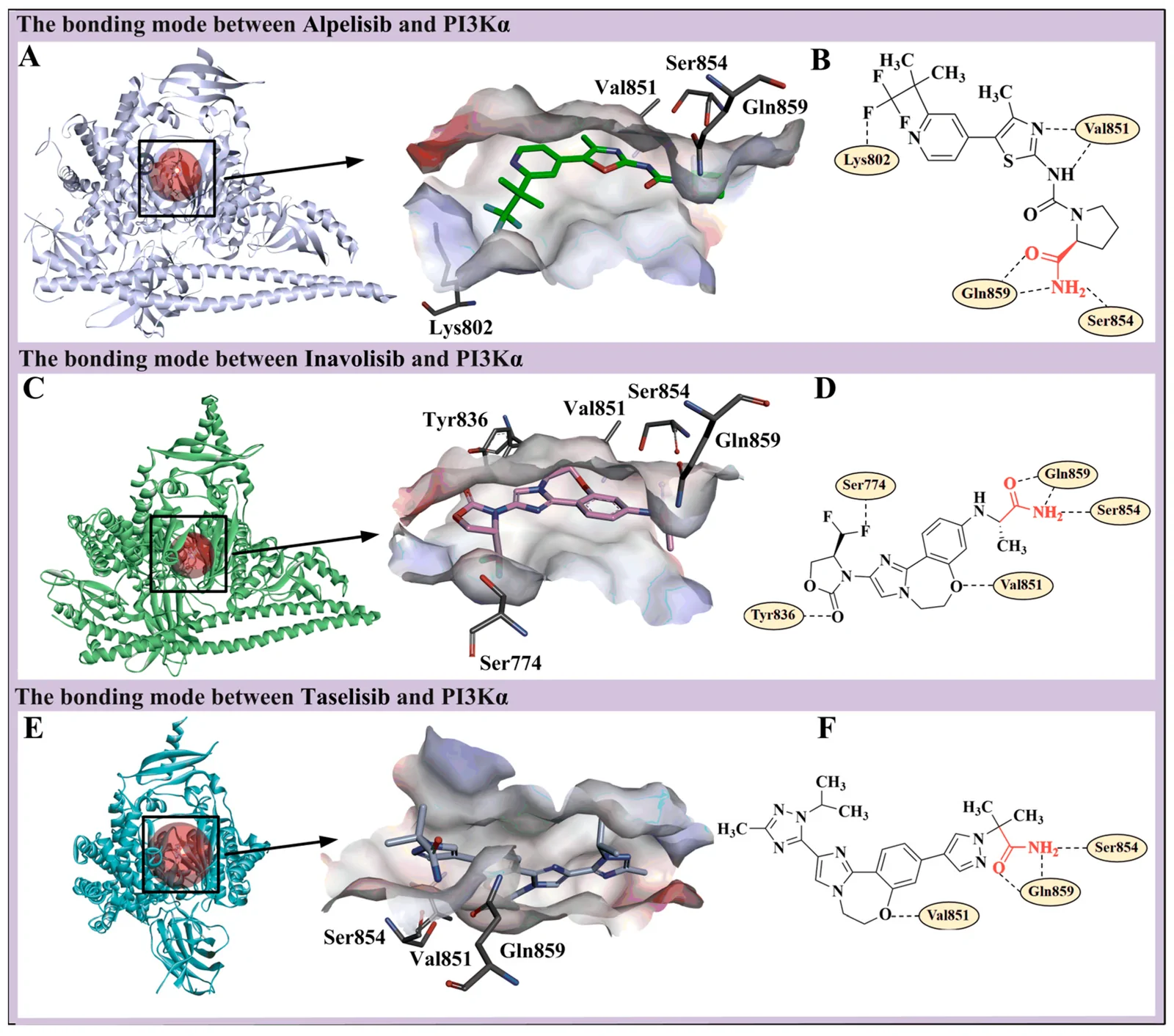
Binding characteristics of ATP-competitive PI3Kα inhibitors with PI3Kα. (**A**) The binding site of alpelisib in PI3Kα; (**B**) Interaction of alpelisib with PI3Kα; (**C**) The binding site of inavolisib in PI3Kα; (**D**) Interaction of inavolisib with PI3Kα; (**E**) The binding site of taselisib in PI3Kα; (**F**) Interaction of taselisib with PI3Kα.

**Figure 6:**
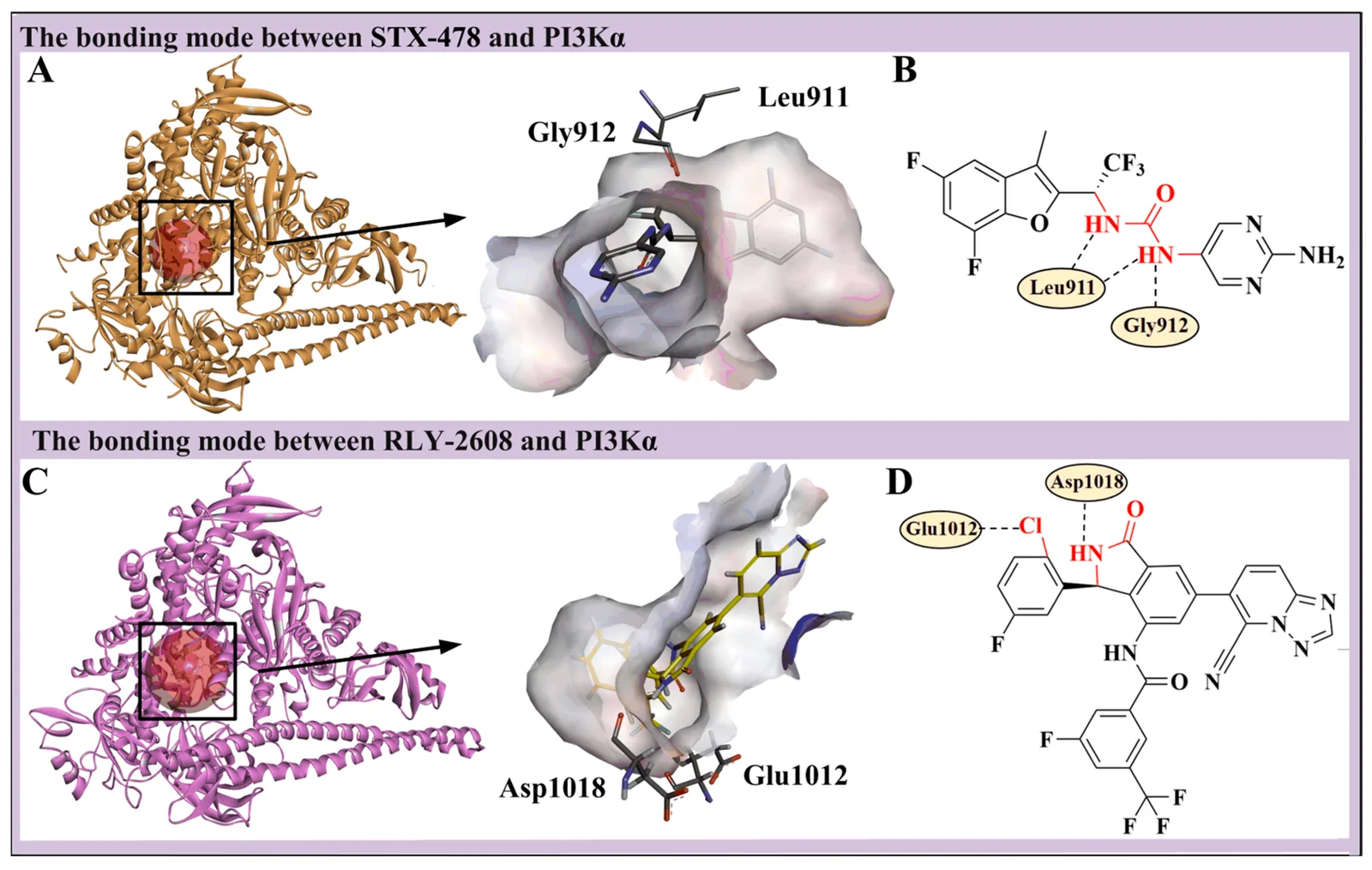
Binding characteristics of allosteric PI3Kα inhibitors with PI3Kα. (**A**) The binding site of STX-478 in PI3Kα; (**B**) Interaction of STX-478 with PI3Kα; (**C**) The binding site of RLY-2608 in PI3Kα; (**D**) Interaction of RLY-2608 with PI3Kα.

**Table 1:** Summary of representative ATP-competitive PI3Kα inhibitors.

Drug (Company)	Disease Indication	IC_50_ (nM)			
		PI3Kα	PI3Kβ	PI3Kδ	PI3Kγ
Alpelisib (Novartis, Switzerland)	Breast cancer; other solid tumors	4.6	1156	290	250
Inavolisib (Genentech, United States)	Breast cancer; other solid tumors	0.034	100	12	18
CYH33 (Shanghai Haihe Biopharma, China)	Solid tumors	5.9	600	79	225
Serabelisib (Takeda, Japan)	Solid tumors	15	4500	13,900	1900
Taselisib (Genentech, United States)	Breast cancer	0.29	9.1	0.12	0.97
CH5132799 (Menarini, Italy)	Breast cancer; colorectal cancer	14	120	500	36

**Table 2:** Overview of clinical trials of alpelisib.

Drug(s)	Disease Indication	Phase	Status	Locations	Identifier
Alpelisib, chemotherapy	Breast cancer	II	Terminated (low recruitment)	France	NCT03386162
Alpelisib	Breast cancer, PROS	Expanded access	Available	No location data	NCT04085653
Alpelisib	Healthy volunteers	I	Completed	USA	NCT05733455
Alpelisib	Lymphangioma	Expanded access	No longer available	USA	NCT03941782
Alpelisib	Vascular anomalies Vascular anomaly TIE2/*PIK3CA* pathway driven vascular anomalies	II	Active, not recruiting	USA	NCT07543822
Alpelisib, placebo, [1-13C] sodium acetate, [6,6-2H2] D-glucose	Insulin resistance	I	Recruiting	USA	NCT06354088
Alpelisib	Healthy volunteers	I	Completed	UK	NCT05195892
Alpelisib	Hepatic impairment	I	Completed	USA	NCT02624557
Alpelisib, paclitaxel	Solid tumor, stomach cancer	I/II	Unknown status	South Korea	NCT04526470
Alpelisib, sacituzumab, govitecan	Breast cancer	I	Active, not recruiting	USA	NCT05143229
Alpelisib	Breast cancer	Observational	Recruiting	South Korea	NCT05293470
Alpelisib, trametinib	Meningioma	I	Recruiting	France	NCT03631953
Alpelisib pill, fulvestrant injection	EC	II	Active, not recruiting	USA	NCT05154487
Alpelisib, olaparib	Metastatic breast cancer	II	Withdrawn (Other—protocol moved to withdrawn)	No location data	NCT05967286
Alpelisib, AUY922	Advanced or metastatic gastric cancer	I	Completed	Multinational	NCT01613950
Alpelisib, capecitabine	Metastatic colorectal cancer	I/II	Unknown status	South Korea	NCT04753203
Alpelisib	Metastatic breast cancer	Observational	Completed	USA	NCT06083038
Alpelisib, everolimus, exemestane	Advanced breast cancer patients, renal cell cancer and pancreatic tumors	I	Completed	Multinational	NCT02077933
Alpelisib	Breast cancer	Not applicable	Unknown status	France	NCT05107388
Alpelisib	Transorally resectable HPV-associated stage I-IVA oropharyngeal cancer	II	Terminated	USA	NCT03601507
Alpelisib, fulvestrant, dapagliflozin	Metastatic breast cancer	II	Unknown status	USA	NCT05025735
Alpelisib, BGJ398	Advanced and metastatic solid tumors	I	Completed	Multinational	NCT01928459
Alpelisib, pembrolizumab	Melanoma (skin cancer), breast cancer, brain metastasases	I	Recruiting	USA	NCT06545682
Alpelisib, cisplatin	Solid tumors	I	Terminated (Funding)	USA	NCT02620839
Alpelisib	PROS	II	Recruiting	Multinational	NCT06997588
Alpelisib, placebo	Lymphatic malformations	II/III	Recruiting	Multinational	NCT05948943
Alpelisib	PROS	Observational	Completed	Multinational	NCT04285723
L-NMMA, alpelisib	HER2^−^ metaplastic breast cancer	II	Recruiting	USA	NCT05660083
Alpelisib	Breast cancer	II	Terminated (Limited amount of eligible participants)	USA	NCT05966584
Alpelisib, fulvestrant	Breast cancer	Observational	Completed	India	NCT05022342
Alpelisib, enzalutamide	Androgen receptor and PTEN positive metastatic breast cancer	I	Completed	USA	NCT03207529
Alpelisib	HR^+^ advanced or metastatic breast cancer	Expanded access	No longer available	No location data	NCT03706573
Alpelisib, carboplatin, hyperpolarized carbon-13 (13C), pyruvate	Solid tumors and HPV-positive squamous cell carcinoma	I	Withdrawn (sponsor funding)	No location data	NCT05472220
Alpelisib	Advanced solid tumor	I	Completed	Japan	NCT01387321
Alpelisib, placebo	PROS	II	Active, not recruiting	Multinational	NCT04589650
AEB071, alpelisib	Uveal melanoma	I	Completed	USA	NCT02273219
Alpelisib, fulvestrant, vinorelbine, capecitabine, eribulin	Advanced breast cancer	III	Active, not recruiting	Multinational	NCT05063786
Alpelisib, matching placebo	Megalencephaly-capillary malformation polymicrogyria syndrome (MCAP)	II	Recruiting	France	NCT05577754
Alpelisib, olaparib, Paclitaxel, pegylated liposomal doxorubicin (PLD)	Ovarian cancer	III	Terminated (sponsor decision)	Multinational	NCT04729387
BMK120, capecitabine, Alpelisib, trastuzumab, lapatinib	Metastatic breast cancer	I	Completed	USA	NCT01300962
LEE011, letrozole, alpelisib	Breast cancer	I/II	Active, not recruiting	Multinational	NCT01872260
LSZ102, LEE011, BYL719	Advanced or metastatic ER^+^ breast cancer	I	Terminated (sponsor decision)	Multinational	NCT02734615
Pioglitazone, alpelisib	Metastatic breast cancer, hyperinsulinism, hyperglycemia drug induced	early I	Recruiting	Israel	NCT05753657
Cisplatin, cetuximab, alpelisib	HPV-associated oropharyngeal squamous cell carcinoma	I/II	Withdrawn	No location data	NCT02298595
Imatinib, alpelisib	Gastrointestinal stromal tumor	I	Completed	Multinational	NCT01735968
Alpelisib, nab-paclitaxel	Anthracycline refractory triple negative breast cancer	II	Terminated (<75% participation)	USA	NCT04216472
Alpelisib, fulvestrant	Advanced breast cancer	IV	Completed	India	NCT05631795
LGX818, cetuximab, alpelisib	Colorectal cancer	II	Completed	Multinational	NCT01719380
Alpelisib	HR^+^ HER2^−^ breast cancer	Observational	Completed	USA	NCT06705504
Tipifarnib, alpelisib	HNSCC	I/II	Completed	USA	NCT04997902
Alpelisib, nab-paclitaxel	Breast cancer	I/II	Completed	USA	NCT02379247
Alpelisib, fulvestrant, canagliflozin	Metastatic breast cancer	II	Active, not recruiting	USA	NCT05090358
Alpelisib	PROS	Observational	Completed	USA	NCT05294289
Palazestrant, ribociclib, alpelisib, Everolimus, atirmociclib	Breast cancer	I	Recruiting	USA, Australia	NCT05508906
LEE011, alpelisib, fulvestrant, BKM120	Breast cancer	I	Completed	Multinational	NCT02088684
Alpelisib, buparlisib, placebo	Breast cancer	II	Completed	Multinational	NCT01923168
Alpelisib, AMG 479	Solid tumors	I/II	Terminated	Multinational	NCT01708161
Alpelisib	Breast cancer	Observational	Completed	Switzerland	NCT05073120
Alpelisib, capecitabine	Rectal cancer	I	Terminated (lack of accural)	USA	NCT02550743
Alpelisib, fulvestrant	Advanced breast cancer	II	Active, not recruiting	Japan	NCT04524000
Alpelisib, alpelisib matching placebo, trastuzumab, pertuzumab	Advanced HER2^+^ breast cancer	III	Active, not recruiting	Multinational	NCT04208178
Alpelisib	Breast cancer	I	Completed	USA	NCT02038010
Alpelisib	PROS	II	Active, not recruiting	Multinational	NCT04980833
Alpelisib	HNSCC	I/II	Terminated (sponsor decision (slow recruitment))	Multinational	NCT01602315
Alpelisib, fulvestrant, aromatase inhibitor	Breast cancer	II	Active, not recruiting	USA	NCT04762979
Alpelisib, fulvestrant	HR^+^ HER2^−^ breast cancer With a *PIK3CA* mutation	Observational	Terminated (low recruitment)	No location data	NCT04967248
Alpelisib, MEK162	Advanced solid tumors	I	Completed	Multinational	NCT01449058
Alpelisib, LJM716, trastuzumab	Metastatic HER2^+^ breast cancer	I	Completed	USA	NCT02167854
Alpelisib, placebo, nab-paclitaxel	Triple negative breast neoplasms	III	Terminated (slow enrollment)	Multinational	NCT04251533
Cetuximab, alpelisib	HNSCC	I	Completed	USA	NCT02282371
Alpelisib	HNSCC	Not applicable	Completed	Canada	NCT03138070
Alpelisib, fulvestrant	Solid tumors	I	Completed	Multinational	NCT01219699
LGH447, alpelisib	Relapsed and refractory multiple myeloma	I	Completed	Multinational	NCT02144038
BYL719, gemcitabine, (nab)-paclitaxel	Pancreatic cancer	I	Completed	USA	NCT02155088
Alpelisib, buparlisib	Breast cancer	I	Completed	Multinational	NCT02058381
Alpelisib, letrozole	HR^+^ metastatic breast cancer	I	Active, not recruiting	USA	NCT01791478
Alpelisib, letrozole, exemestane	Metastatic or locally-advanced unresectable breast cancer	I	Completed	USA	NCT01870505
Alpelisib, metformin, fulvestrant, letrozole, exemestane, vildagliptin, tamoxifen	Breast cancer	II	Completed	Spain	NCT04300790
Alpelisib, fulvestrant	Breast cancer	II	Terminated (slow recruitment)	USA, Malaysia	NCT04899349
Alpelisib, paclitaxel	Neoplasms, breast neoplasms, head and neck neoplasms	I	Completed	Multinational	NCT02051751
Alpelisib, fulvestrant, placebo	Breast neoplasms	II	Active, not recruiting	China	NCT04544189
LJM716, alpelisib, paclitaxel, docetaxel, irinotecan	Esophageal squamous cell carcinoma	I	Completed	Multinational	NCT01822613
Alpelisib, fulvestrant, goserelin	Advanced breast cancer	II	Terminated (low enrollment)	Sweden	NCT04862143
Alpelisib, cisplatin	HNSCC	I	Completed	Canada	NCT02537223
fulvestrant, alpelisib, placebo	Breast cancer	III	Completed	Multinational	NCT02437318
Alpelisib, fulvestrant	HR^+^/HER2^−^ advanced or metastatic breast cancer	Observational	Completed	Ireland	NCT05853432
Alpelisib, fulvestrant	Advanced luminal breast cancer	II	Active, not recruiting	Netherlands	NCT05392608
Alpelisib, olaparib	Breast cancer	I	Completed	USA	NCT01623349
Alpelisib, tucatinib, fulvestrant	Breast cancer	I/II	Active, not recruiting	USA	NCT05230810
Alpelisib	Breast cancer	Observational	Completed	Russia	NCT04943497
Alpelisib, INC280, LDK378, MEK162	Adenocarcinoma lung cancer; squamous cell lung carcinoma	II	Completed	China	NCT02276027
Alpelisib, fulvestrant, alpelisib-matching placebo	Breast cancer	III	Active, not recruiting	Multinational	NCT05038735
Alpelisib	Metastatic breast cancer	II	Completed	Australia	NCT02506556
Ribociclib, letrozole, alpelisib, fulvestrant	Breast cancer	III	Completed	Italy	NCT03439046
Alpelisib, ribociclib	Breast cancer stage IV	II	Active, not recruiting	France	NCT05625087
Alpelisib, fulvestrant, letrozole, goserelin, leuprolide	Breast cancer	II	Completed	Multinational	NCT03056755
Inavolisib, fulvestrant, alpelisib, Bupropion, omeprazole, midazolam	Breast cancer	III	Active, not recruiting	Multinational	NCT05646862
Alpelisib	Recurrent or metastatic HNSCC	II	Unknown status	South Korea	NCT02145312
BYL719, poziotinib, nintedanib, Abemaciclib, durvalumab, tremelimumab	HNSCC, head and neck neoplasms	II	Completed	South Korea	NCT03292250

**Table 3:** Overview of clinical trials of inavolisib.

Drug(s)	Disease Indication	Phase	Status	Locations	Identifier
Inavolisib, atezolizumab	*PIK3CA*-mutated cancers	I	Active, not recruiting	Multinational	NCT06496568
Inavolisib	EC	II	Not yet recruiting	Italy	NCT07522697
Inavolisib	Breast cancer	Observational	Not yet recruiting	No location data	NCT07543250
Inavolisib, enzalutamide, abiraterone, docetaxel	Metastatic castration-resistant prostate cancer	II	Recruiting	Multinational	NCT07287150
Inavolisib, ribociclib, letrozole	Breast cancer	II	Recruiting	Multinational	NCT07054190
Inavolisib, fulvestrant	Breast cancer	II	Recruiting	Multinational	NCT07368998
Inavolisib, PHESGO, endocrine therapy	Breast cancer	II	Recruiting	Germany, Italy	NCT05306041
Inavolisib	Hepatic impairment	I	Recruiting	USA	NCT07144111
Inavolisib, eribulin	Breast cancer	II	Not yet recruiting	No location data	NCT07441512
Inavolisib	Breast cancer	II	Not yet recruiting	No location data	NCT07245797
Inavolisib, palbociclib, fulvestrant	Locally advanced breast cancer, metastatic breast cancer	Observational	Recruiting	China	NCT07347600
Inavolisib, ribociclib, fulvestrant	Breast cancer	II	Not yet recruiting	No location data	NCT07618390
Inavolisib, bevacizumab, FOLFOX, FOLFIRI, placebo	Colorectal cancer	II	Withdrawn (sponsor’s decision)	No location data	NCT07323576
Inavolisib, ribociclib, fulvestrant	Breast cancer	II	Recruiting	Multinational	NCT07405801
Inavolisib, palbociclib, fulvestrant	Breast cancer	II/III	Active, not recruiting	Multinational	NCT04191499
Inavolisib	Breast cancer, solid tumor	I	Active, not recruiting	Multinational	NCT03006172
Inavolisib, phesgo	Metastatic breast cancer	III	Recruiting	Multinational	NCT05894239
Inavolisib, CDK4/6 inhibitor, letrozole	Breast cancer	III	Recruiting	Multinational	NCT06790693
Inavolisib, fulvestrant	Breast cancer	III	Active, not recruiting	Multinational	NCT05646862

**Table 4:** Overview of clinical trials of CYH33.

Drug(s)	Disease Indication	Phase	Status	Locations	Identifier
CYH33	Advanced solid tumors	I	Completed	China	NCT03544905
CYH33, fulvestrant, letrozole, palbociclib	Advanced breast cancer	I	Unknown status (not yet recruiting)	No location data	NCT04856371
CYH33, placebo	PROS, *PIK3CA*-related vascular malformations (PRVM)	I/II	Recruiting	China	NCT06975618
CYH33	Ovarian cancer, recurrent cancer	II	Active, not recruiting	China	NCT05043922
CYH33	Advanced solid tumors	I	Terminated (business decision)	USA, China, Australia	NCT04586335

**Table 5:** Overview of clinical trials of serabelisib.

Drug(s)	Disease Indication	Phase	Status	Locations	Identifier
Serabelisib, sapanisertib, fulvestrant	HR^+^ HER2^−^ breast cancer	I/II	Recruiting	USA	NCT07558733
Serabelisib	Metastatic solid tumors	I	Completed	USA, UK, Spain	NCT01449370
Serabelisib, canagliflozin	Advanced solid tumors	I/II	Unknown status (not yet recruiting)	No location data	NCT04073680
Paclitaxel, sapanisertib, serabelisib	Advanced solid tumor	I	Completed	USA	NCT03154294
Sapanisertib, serabelisib, paclitaxel	EC	II	Recruiting	USA	NCT06463028
Serabelisib, nab paclitaxel	Advanced solid tumor	I	Terminated (program prioritization)	USA	NCT05300048
Serabelisib, lansoprazole	Neoplasm, advanced or metastatic	I	Completed	No location data	NCT02625259
Sapanisertib, serabelisib	Advanced nonhematologic malignancies	I	Completed	USA, UK, Spain	NCT01899053
Docetaxel, serabelisib	Non-small cell lung cancer	I/II	Terminated (lack of efficacy in combination)	USA, Canada, Netherlands	NCT02393209
Paclitaxel, sapanisertib, serabelisib	Endometrial neoplasms	II	Completed	Multinational	NCT02725268
Sapanisertib, serabelisib	Triple negative breast cancer	II	Completed	USA	NCT03193853
Serabelisib, TAK-659, alisertib, paclitaxel, docetaxel	Advanced and metastatic gastric or gastroesophageal adenocarcinoma	I	Terminated (business decision; no safety or efficacy concerns)	USA, Spain	NCT02551055
Everolimus, sapanisertib, serabelisib	Clear-cell metastatic renal cell carcinoma	II	Completed	Multinational	NCT02724020

**Table 6:** Overview of clinical trials of taselisib.

Drug(s)	Disease Indication	Phase	Status	Locations	Identifier
Taselisib	PROS	I/II	Terminated (safety reasons)	France	NCT03290092
Enzalutamide, taselisib	Metastatic breast cancer	I/II	Terminated (interim analysis—toxicity)	USA	NCT02457910
Taselisib, trastuzumab, emtansine, pertuzumab, trastuzumab, paclitaxel	Breast cancer	I	Completed	USA	NCT02390427
Taselisib	Advanced lymphoma, Advanced malignant solid neoplasm, Hematopoietic and lymphoid cell neoplasm, Refractory lymphoma, Refractory malignant solid neoplasm, Refractory multiple myeloma	II	Active, not recruiting	USA	NCT04439175
Taselisib	Squamous cell lung carcinoma	II	Completed	USA	NCT02785913
Letrozole, taselisib	Breast cancer	II	Completed	Multinational	NCT02273973
Taselisib, placebo, fulvestrant	Breast cancer	III	Terminated (modest clinical benefit and limited tolerability)	Multinational	NCT02340221
Palbociclib, taselisib, pictilisib	Advanced solid tumors, Breast cancer	I	Completed	UK	NCT02389842

**Table 7:** Summary of representative allosteric PI3Kα inhibitors.

Drug (Company)	Disease Indication	IC_50_ (nM) of PI3Kα			
		WT	H1047R	E542K	E545K
STX-478 (Scorpion Therapeutics)	Solid tumors	131	9.4	113	71
RLY-2608 (Relay Therapeutics)	Solid tumors	48	4	-	-

**Table 8:** Overview of clinical trials of RLY-2608, LOXO-783, STX-478.

Drug(s)	Disease Indication	Phase	Status	Locations	Identifier
RLY-2608, placebo	PROS, MCAP, lymphatic malformations, vascular malformations, *PIK3CA* mutation, CLOVES syndrome, klippel trenaunay syndrome	II	Recruiting	Multinational	NCT06789913
RLY-2608, capivasertib, fulvestrant	Breast cancer	III	Recruiting	Multinational	NCT06982521
RLY-2608, fulvestrant, palbociclib, PF-07220060	Breast cancer	I	Recruiting	Multinational	NCT05216432
STX-478	Healthy volunteer	I	Completed	USA	NCT06901336
STX-478, placebo, ribociclib, palbociclib, abemaciclib, anastrozole, letrozole, exemestane, fulvestrant	Breast neoplasms, neoplasm metastasis	III	Recruiting	Multinational	NCT07174336
STX-478, fulvestrant, ribociclib, palbociclib, letrozole, anastrozole, exemestane, tamoxifen, abemaciclib, imlunestrant, metformin	Breast cancer, solid tumors	I/II	Recruiting	Multinational	NCT05768139
LOXO-783, cholestyramine	Healthy volunteer	I	Completed	USA	NCT05894928
LOXO-783	Healthy volunteer	I	Completed	USA	NCT06102512
LOXO-783	Healthy volunteer	I	Completed	USA	NCT06028425
LOXO-783, fulvestrant, imlunestrant, abemaciclib, paclitaxel, anastrozole, exemestane, or letrozole	Breast cancer	I	Active, not recruiting	Multinational	NCT05307705

## Data Availability

No new data were created or analyzed in this study. Data sharing is not applicable to this article.

## Supplementary Materials

The following supporting information can be downloaded at: https://www.mdpi.com/article/10.3390/cimb48100968/s1.

## Abbreviations

The following abbreviations are used in this manuscript:

PIP2	Phosphatidylinositol 4,5-bisphosphate
PIP3	Phosphatidylinositol 3,4,5-trisphosphate
ABD	N-terminal adaptor binding domain
RBD	Ras binding domain
HR^+^	Hormone receptor-positive
HER2^−^	Human epidermal growth factor receptor 2-negative
HRR	Homologous recombination repair
PFS	Progression-free survival
PR	Partial response
SD	Stable disease
HNSCC	Head and neck squamous cell carcinoma
HPV	Human papillomavirus
IC_50_	Half-maximal inhibitory concentration
PROS	*PIK3CA*-related overgrowth spectrum
MCAP	Megalencephaly-capillary malformation polymicrogyria syndrome
EC	Endometrial carcinoma
PRVM	*PIK3CA*-related vascular malformations
FBG	Fasting blood glucose
PROTACs	Proteolysis-targeting chimeras
DACs	Degrader-antibody conjugates
PI3Kα	Phosphatidylinositol 3-kinase α
mTOR	Mammalian target of rapamycin
PTEN	Phosphatase and tensin homolog
SERD	Selective estrogen receptor degrader
Akt	Protein kinase B
PDK1	3-phosphoinositide-dependent protein kinase 1
mTORC1/2	Mechanistic target of rapamycin complex 1/2
p70S6K	p70 ribosomal S6 kinase
TSC2	Tuberous sclerosis complex 2
PRAS40	Proline-rich Akt substrate of 40 kDa
4EBP1	Eukaryotic translation initiation factor 4E-binding protein 1
MAPK	Mitogen-activated protein kinase
ERK	Extracellular signal-regulated kinase
GSK3	Glycogen synthase kinase-3
FOXO	Forkhead box O
NFκB	Nuclear factor kappa B
MDM2	Murine double minute 2
NGS	Next-generation sequencing
ctDNA	Circulating tumor DNA
SGLT2	Sodium-glucose cotransporter 2
