# Supplementary material for: Targeting PI3Kα with Small Molecules: Progress and Challenges in Tumor Treatment
Source: Curr Issues Mol Biol. 2026 Sep 22;48(10):968. doi: 10.3390/cimb48100968 (PMC13703482; doi:10.3390/cimb48100968)
Supplement: Supplementary file 1 [file cimb-48-00968-s001.zip › cimb-4545101-supplementary.pdf]

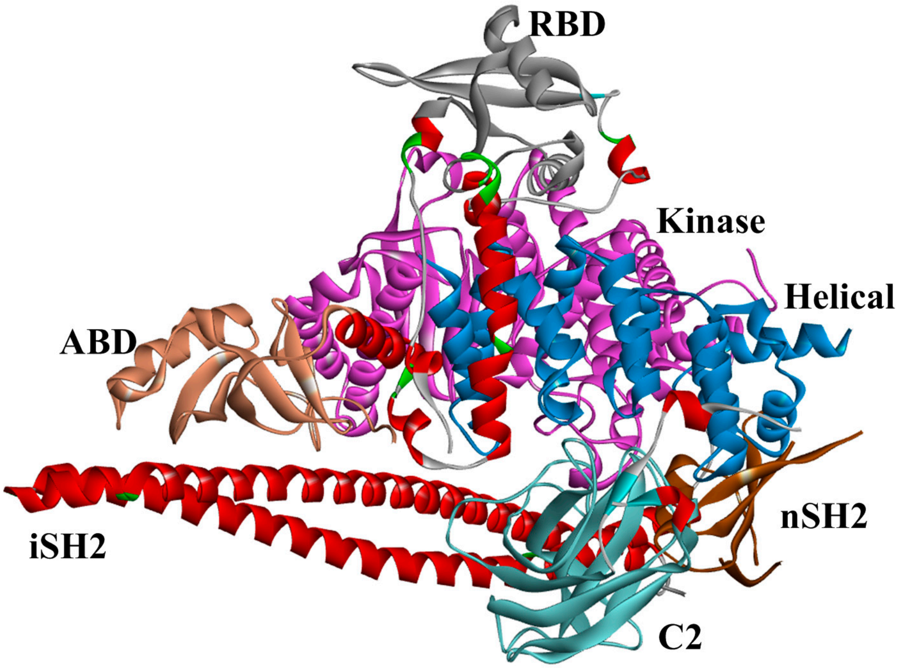

Figure S1. The p110α/niSH2 heterodimer. Orange: ABD; gray: RBD; light blue: C2; dark blue: helical; purple: kinase domain; red: iSH2; brown: nSH2.
